# Comprehensive and Computable Molecular Diagnostic Panel (C2Dx) From Small Volume Specimens for Precision Oncology: Molecular Subtyping of Non-Small Cell Lung Cancer From Fine Needle Aspirates

**DOI:** 10.3389/fonc.2021.584896

**Published:** 2021-04-16

**Authors:** Jing Su, Lynn S. Huang, Ryan Barnard, Graham Parks, James Cappellari, Christina Bellinger, Travis Dotson, Lou Craddock, Bharat Prakash, Jonathan Hovda, Hollins Clark, William Jeffrey Petty, Boris Pasche, Michael D. Chan, Lance D. Miller, Jimmy Ruiz

**Affiliations:** ^1^ Department of Cancer Biology, Wake Forest School of Medicine, Winston-Salem, NC, United States; ^2^ Department of Biostatistics and Health Data Science, Indiana University School of Medicine, Indianapolis, IN, United States; ^3^ Department of Biostatistics and Data Science, Wake Forest School of Medicine, Winston-Salem, NC, United States; ^4^ Department of Pathology, Wake Forest School of Medicine, Winston-Salem, NC, United States; ^5^ Department of Medicine (Pulmonology and Critical Care), Wake Forest School of Medicine, Winston-Salem, NC, United States; ^6^ Department of Radiation Oncology, Wake Forest School of Medicine, Winston-Salem, NC, United States; ^7^ Department of Radiology, Wake Forest School of Medicine, Winston-Salem, NC, United States; ^8^ Department of Medicine (Hematology & Oncology), Wake Forest School of Medicine, Winston-Salem, NC, United States; ^9^ W.G. (Bill) Hefner Veteran Administration Medical Center, Cancer Center, Salisbury, NC, United States

**Keywords:** non-small cell lung cancer, molecular signature, fine needle aspiration, logistic regression, elastic net regularization, cancer subtyping

## Abstract

The *Comprehensive, Computable NanoString Diagnostic gene panel* (C2Dx) is a promising solution to address the need for a molecular pathological research and diagnostic tool for precision oncology utilizing small volume tumor specimens. We translate subtyping-related gene expression patterns of Non-Small Cell Lung Cancer (NSCLC) derived from public transcriptomic data which establish a highly robust and accurate subtyping system. The C2Dx demonstrates supreme performance on the NanoString platform using microgram-level FNA samples and has excellent portability to frozen tissues and RNA-Seq transcriptomic data. This workflow shows great potential for research and the clinical practice of cancer molecular diagnosis.

## Background

In this era of precision oncology, there has been a rapid adoption of targeted therapy for detected driver mutations in non-small-cell lung cancer (NSCLC). As a result, there is a growing tension between the increasing tissue demands required for comprehensive molecular diagnosis and the traditional, immunohistochemical and morphology-based lung cancer subtyping approaches. Currently, the treatment of NSCLC is dependent on accurate histological and molecular subtyping as well as other clinical and pathological features (1, 2). Clinical guidelines such as the NCCN (the National Comprehensive Cancer Network) guidelines (Version 5.2018) (3) and the 2015 World Health Organization classification (4) recommend that histologic and molecular features be used in determining treatment options. Pathologists use hematoxylin-eosin (H&E)-staining on samples to identify NSCLC subtypes like adenocarcinoma (LUAD) and squamous cell carcinoma (LUSC). Immunohistochemical stains are used to diagnose subtype, but in the event further stains are unable to do so, a diagnosis of “NSCLC, not otherwise specified (NOS)” is made. Positive staining for thyroid transcription factor 1 (TTF1, coded by gene NKX2-1) and napsin A (novel aspartic proteinase of the pepsin family) help to classify LUAD. LUSC stains for markers tumor protein p63, and cytokeratins (CK) 5, 6, and 7. After lung cancer subtyping is complete, non LUSC are evaluated for targetable driver genomic alterations such as epidermal growth factor receptor (EGFR) mutations, anaplastic lymphoma kinase (ALK) rearrangements, c-Ros oncogene 1 (ROS1) rearrangements, and BRAF V600E mutation. Checkpoint markers such as PD-1/PD-L1 (programmed cell death-1 and programmed death-ligand 1) (1) are further examined in all subtypes of NSCLC. This process determines the treatment options for patients.

In advanced and metastatic NSCLC cases, small volume biopsies are generally obtained through the least invasive diagnostic option. The morphology-based subtyping can utilize a significant amount of limited tissue and can lead to insufficient biopsy material for molecular testing. Despite the fast-growing quantity demands for diagnostic tissues, minimally invasive small volume biopsies are commonly utilized for diagnosis. The fine-needle aspirate (FNA) is now the most routinely utilized biopsy approach for NSCLC (5–7). While sufficient for confirming the existence of cancers through transthoracic needle biopsy (TTNB), the amount of cellular material from FNAs can be sparse. This is often cited (8) as the limiting factor to the correct classification of NSCLC histology, a problem further compounded by the poor cellular differentiation characteristic of advanced-stage disease.

Over the last decade, we have been introduced to precision oncology initiatives (9) and novel immunotherapy treatment options that require additional biopsy tissue for molecular testing and next generation sequencing (10, 11). New and emerging therapies on or off clinical study require additional tissues for examination and continues to contribute to the growing demand on limited tissues obtained at diagnosis. In real-world clinical settings, small tumor sample volume limits the implementation of many next-generation sequencing (NGS) technologies (12). For example, approximately 14% of clinical FNA samples qualified for the 324-gene FoundationOne CDx targeted genomics sequencing (13–15) after the samples were used for diagnosis and molecular profiling. NGS devices that are specially designed for small sample volumes, such as the 26-gene Oncomine Dx Target Test, can only test a small number of genes. Liquid biopsy technologies, though available in NSCLC patients (16, 17), are only utilized if tissue samples are exhausted and additional specimens cannot be obtained. Therefore, greater effort and novel strategies are required to minimize consumption of diagnostic tissues in order to maximize diagnostic output.

We have been pioneering the development of a novel device, *the Comprehensive, Computable NanoString Diagnostic gene panel* (C2Dx), as a promising solution to address the burning clinical need in molecular pathological diagnosis with small volume tumor specimens. The NanoString nCounter^®^ (18) is a digital multiplexed molecular diagnostic platform supporting the molecular profiling of up to 800 genes in a single panel. It uses extremely small amounts of tissues and has been used successfully in precision oncology applications (19, 20), including the detection of fusion genes (21) in lung cancer cytology and molecular subtyping of diffuse large B-cell lymphoma (22). We established clinical and laboratory protocols for reliable lung cancer RNA acquisition from a single transbronchial or transthoracic needle pass using fine needle aspiration (23). Key differentially expressed genes in LUAD and LUSC cases demonstrated high consistency across the NanoString platform using FNA samples and the RNA-Seq platform using the bulk tumor samples. We have also demonstrated the successful translation of knowledge learned from bulk-based transcriptomics data to a NanoString gene panel in exploring novel prognostic molecular subtypes in appendiceal mucinous neoplasms (24). A critical step from what we have achieved toward a clinical C2Dx device is to transfer knowledge discovered from existing omics data to a highly robust computable gene panel. In this work, we used the NSCLC subtyping task as a working example to demonstrate the feasibility of such quantitative translational modeling from bulk transcriptomic data to FNA-based NanoString gene panels.

**Figure 1:**
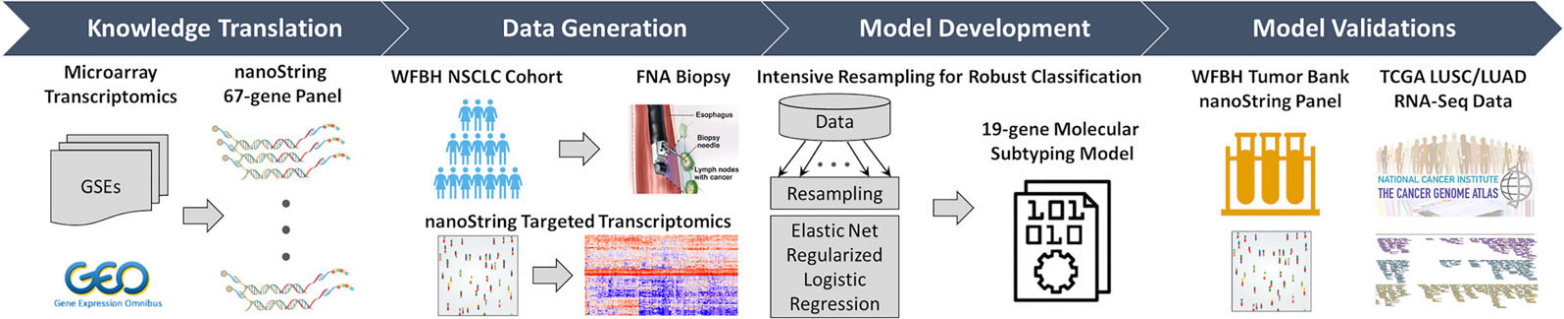
Overall workflow of the C2Dx development. The development of the C2Dx NSCLC subtyping device was composed of the following four steps: 1. Knowledge translation. Four microarray-based transcriptomics datasets of NSCLC cases were extracted from Gene Expression Omnibus (GEO) and used to identify 67 subtyping-related genes, including 23 LUAD genes, 40 LUSC genes, and 4 housekeeping genes. A corresponding nanoString gene panel was established. 2. Data generation. Targeted transcriptomics data were generated using the nanoString 67-gene panel for 83 FNA biopsy samples collected from WFBH NSCLC patients, including 47 LUAD, 25 LUSC, and 11 NOS cases. 3. Model development. Intensive resampling strategy was used in elastic-net regularized logistic regression to develop the final 19-gene molecular subtyping model. 4. Model validation. The developed subtyping model was validated using the transcriptomics data of the 19 genes collected from frozen tissue from WFBH’s tumor bank using nanoString platform and from the TCGA’s RNA-Seq data generated from bulk tissues.

In this work, we developed a highly robust, high performing, low tissue demanding, molecular subtyping system to compliment histology determination (**Figure 1**). Using a 67-gene NanoString transcriptomic gene panel learned from four existing NSCLC omics datasets and generated from nanogram-level RNA samples, we implemented an intensive resampling strategy for robust elastic net regularization of logistic regression. We established a NanoString-based 19-gene panel (15 signature genes and 4 housekeeping genes) and demonstrated a competitive NSCLC subtyping classification with sound accuracy when compared to interpretations from experienced pathologists. We were able to utilize a single FNA pass to determine adenocarcinoma from squamous histology. This strategy potentially allows for minimal diagnostic tissue utilization for subtyping which can lead to a decrease rate of tissue depletion and need for additional invasive biopsies. The 19-gene subtyping model demonstrates high accuracy across different sample types (FNA specimens and frozen tumor tissues) and transcriptomic platforms (NanoString gene panels and RNA-seq). This work proved the concept of transferring knowledge from omics data generated at bench-side to a robust computable device implementable at bedside through robust translational modeling and FNA-based NanoString C2Dx system.

## Methods

### Overview of the Pipeline

The aim of this study is to establish a robust, comprehensive, computable molecular subtyping device using FNA-based nanoString gene panel and knowledge of subtype-related gene expression from public data to distinguish LUAD and LUSC in NSCLC cases. As demonstrated in **Figure 1**, as previously reported (23), knowledge about LUAD/LUSC subtyping related genes was learned from a meta-cohort (n = 490) microarray transcriptomics data extracted from public source [Gene Expression Omnibus: GSE10445 (25), GSE4573 (26), and GSE3141 (27); the Director’s Challenge Consortium for the Molecular Classification of Lung Adenocarcinoma (28)] and translated to a nanoString 67-gene panel for clinical use. This panel was used to generate targeted transcriptomics data on tissues collected by FNA-based biopsy from 83 WFBH’s NSCLC patients. A robust 19-gene molecular subtyping model were trained using the generated data, intensive resampling, and elastic-net regularized logistic regression. The molecular phenotyping model was validated using nanoString-generated data of 44 frozen samples collected from WFBH’s tumor tissue bank and RNA-Seq data of 1,016 samples from TCGA’s LUAD and LUSC cohorts.

### Classifier Development

Four publicly available lung cancer cohorts were used for 67-gene panel construction, including the DUKE (n = 111, GEO: GSE3141), PARIS (n = 74, GEO: GSE10445), UM (n = 13, GEO: GSE4573), and the DC (n = 442, NCI caArray: Jacob-00182).

### Patient Selection and Procedures

The study was approved by the Institutional Review Board of the Wake Forest Baptist Medical Center from 2013 and 2015. Patients completed informed consent forms prior to enrollment. Eligibility requirements were for radiographic evidence for lung cancer or have a previously diagnosed NSCLC with potential recurrence requiring re-biopsy.

Patients enrolled on study agreed to provide one additional small volume aspirate pass for RNA collection after completion of standard of care diagnostic tissue collection for presumed NSCLC. Patients underwent FNA of primary or secondary metastatic lesions. Exclusion criteria included patients whose FNA biopsy was unable to provide subtype classification by pathology, consistent with small cell carcinoma, non-malignant etiologies, or returned non-diagnostic. Small volume FNA diagnostic modalities were obtained by: endobronchial ultrasound guided transbronchial needle aspiration (EBUS-TBNA), conventional trans-bronchial needle aspiration (cTBNA) and trans-thoracic needle biopsy (TTNB). Bronchoscopy cases were performed by pulmonogists using rapid on-site cytology evaluation (ROSE) to help decipher results. TTNB were performed by interventional radiologists with training and experience with CT-guided thoracic procedures.

### Model Development

For developing the molecular subtyping model, we established a WFBH-FNA cohort by collected 83 FNA samples (1.645 ± 2.298 μg RNA) from tumor or lymph node tissues of the Wake Forest Baptist Hospital (WFBH) non-small cell lung cancer patients, including 47 adenocarcinoma (LUAD), 25 squamous cell carcinoma (LUSC), and 11 non-small cell lung cancer but not otherwise specified (NOS) cases. Two cohorts were used for model validation: 1) WFBH-TB. We collected 44 frozen tissue samples (LUAD: 21; LUSC: 23) from WFBH Tissue Bank for internal validation; 2) TCGA. The TCGA RNA-Seq dataset (sample size: 1,145; version: Data Release 8.0; data source: Genomic Data Commons (GDC); data extraction date: September 7, 2017) of 594 LUAD and 551 LUSC samples was used for external validation.

Histology classification of the 127 WFBH samples was determined through diagnostic pathological review of Haemotoxylin and Eosin (H&E) stained microscopic slides of tumor tissues by two mutually blinded pathologists and discordant cases were labeled as NOS.

### NanoString-Based Transcriptomic Data Generation

A 67-gene panel has been constructed and the nanoString-based transcriptomic data has been generated for the study cohort as previously reported (23). Among these genes, 23 were LUAD-associated, 40 were LUSC associated, and 4 were housekeeping genes for normalization purpose. The full gene list can be found in **Supplement Table S6**.

### Individual Normalization of Gene Expression

For NanoString-based FNA data, the raw Reporter Code Count data was used. For TCGA samples, the expressions of the 67 genes in the form of FPKM-UQ (fragments per kilobase per million reads, upper quartile normalized) (29) were used. For each sample, original expression of all genes were log2 transformed, normalized against the mean of the 4 housekeeping genes, and z-transformed. Cross-sample normalization was avoided to simplify clinical implementation. Details are provided in Supplement: Data Processing.

### Logistic Classification Model

Assuming that the subtypes LUAD and LUSC followed a binomial distribution, the odd of the probability of a given sample *i* was from an LUAD patient (*p*) vs. from an LUSC patient (1 - *p*) was modeled using a logistic regression model given by:

$$\log\frac{p_{i}}{1-p_{i}}=\beta_{0}+g_{i}\beta^{T}$$

where ***g_i_*** ≡ [*g_i,1_*, *g_i,2_*, …, *g_i,m_*] was the expression levels of the *m* genes of sample *i*, and *β_0_* and ***β*** ≡ [*β_1_*, *β_2_*, …, *β_m_*] were the regression coefficients.

### Feature Selection and Model Training

Elastic net regularization (30) and cross-validation with intensive resampling was used for feature selection. We first determined optimal model complexity, that is, the optimal number of genes in the model. Let *y_i_* indicate the true subtype of sample *i*, *y_i_* = 1 for LUAD and *y_i_* = 0 for LUSC, the objective function for the elastic net penalized logistic regression model was:

$$\underset{\beta_{0},\beta }{\text{argmin}}\frac{1}{n}\sum_{i=1}^{n}\left[y_{i}-\left(\beta_{0}+g_{i}\beta^{T}\right)-\log\left(1+e^{\beta_{0}+g_{i}\beta^{T}}\right)\right]+\lambda \left[\frac{(1-\alpha ){||\beta ||}^{2}}{2}\right]+\alpha {||\beta ||}_{1}$$

where *n* was the sample size, *λ* > 0 was the tuning factor, 0 ≤ *α* ≤ 1 was the elastic net mixing factor that controlled the mixing percentage between the ridge regularization (*α* = 0) and the LASSO regularization (*α* = 1), ||•||_1_ and ||•||^2^ represented the L1 and L2 norm, respectively. To reach a robust solution, 3-fold cross validation was used for 1,000 resampling iterations with *α* screened from 0 to 1 at step size of 0.1. Evaluating the model performance by accuracy, the 15-signature-gene model was selected with estimated accuracy of 0.902 ± 0.053. All FNA samples was then used to train the final 15-signature-gene classification model using logistic regression with elastic net regularization. **Supplement Figure S2** provided more details on elastic net feature selection. R (version: 3.3.1) packages glmnet (version 2.0-10) (31) and caret (version 6.0-77) (32) was used for feature selection and model training.

### Model Validation

The classification model, with authentic coefficients, was directly implemented on the internal (44 NanoString samples from frozen tumor tissues) and external (1,016 RNA-Seq data from TCGA datasets) to classify TCGA sample subtypes for evaluating model performance.

### Profiling NOS Cases

The 11 FNA samples diagnosed as NOS were analyzed with the 19-gene molecular subtyping model and compared with the LUAD and LUSC cases. The logistic probability patterns of NOS, LUAD, and LUSC cases were analyzed using enrichment analysis and Kolmogoro-Smirnov test.

### Statistics Methods

The convergence of model stability during elastic net regularization with respect to resampling rounds was measured by the estimated values as well as the corresponding 95% confidence intervals of the model complexity, accuracy, and elastic net mixing factor *α*. The receiver operating characteristic (ROC) curves were generated for model performance on the FNA, internal tissue bank, and external TCGA cohorts and the corresponding concordance statistic (c-statistic, a.k.a. the area under the ROC curve, AUC) (33–35) were calculated for model performance comparison.

## Results and Discussion

### Patient Characteristics

Patients’ demographics and clinical characteristics were obtained for the Exploring, Training, and Validation cohorts summarized in **Table 1** and **Supplement Table S1**. Most characteristics were consistent across all cohorts. Such consistency between the research cohorts (Exploring and TCGA) and the clinical cohorts (WFBH-FNA and WFBH-TB) were important for knowledge translation from scientific studies to clinical applications.

**Table 1:** Patients’ characteristics.

Characteristics	Exploring	Training	Validation	
		WFBH-FNA	WFBH-TB	TCGA
Overall Cohort Size	490	83	42	1,016
Age, mean (sd)	65.1 (10.2)	65.9 (9.5)	65.7 (7.7)	66.7 (9.4)
Gender, n (%)				
Female	218 (44.5%)	50 (60.2%)	18 (40.9%)	406 (40.0%)
Male	272 (55.5%)	33 (39.8)	26 (59.1%)	610 (60.0%)
Race, n (%)	—			
Caucasian		72 (86.8%)	38 (86.4%)	738 (72.6%)
African American		9 (10.8%)	5 (11.4%)	82 (8.1%)
Others		2 (2.4%)	1 (2.2%)	196 (19.3%)
Adenocarcinoma	384 (78.4%)	47 (56.6%)	20 (47.7%)	515 (53.7%)
Age, mean (sd)	64.1 (10.3)	65.2 (9.5)	65.3 (9.0)	65.7 (10.0)
Gender, n (%)				
Female	180 (46.9%)	22 (46.8%)	10 (47.6%)	276 (53.6%)
Male	204 (53.1%)	25 (53.2%)	11 (52.4%)	239 (46.4%)
Race, n (%)	—			
Caucasian		40 (85.1%)	19 (90.5%)	389 (75.5%)
African American		6 (12.8%)	2 (9.5%)	52 (10.1%)
Others		1 (2.1%)	0	74 (14.4%)
Squamous Cell Carcinoma	106 (21.6%)	25 (30.1%)	22 (52.3%)	501 (46.3%)
Age, mean (sd)	68.6 (9.1)	66.2 (8.6)	66.0 (6.5)	67.7 (8.6)
Gender, n (%)				
Female	38 (35.8%)	6 (24%)	8 (34.8%)	130 (25.9%)
Male	68 (64.2%)	19 (76%)	15 (65.2%)	371 (74.1%)
Race, n (%)	—			
Caucasian		21 (84%)	19 (82.6%)	349 (69.7%%)
African American		3 (12%)	3 (13.0%)	30 (6.0%)
Others		1 (4%)	1 (4.4%)	122 (24.4%)
NOS	—	11 (13.3%)	—	—

The demographical and clinical characteristics of the 3 cohorts in this study were summarized. WFBH-FNA: the training cohort with tumor tissues collected from FNA biopsy of WFBH patients. WFBH-TB and TCGA: the validation cohorts with frozen tumor tissues collected from surgeries of WFBH patients and TCGA patients, respectively.

### Feature Selection and Model Training

**Figure 2:**
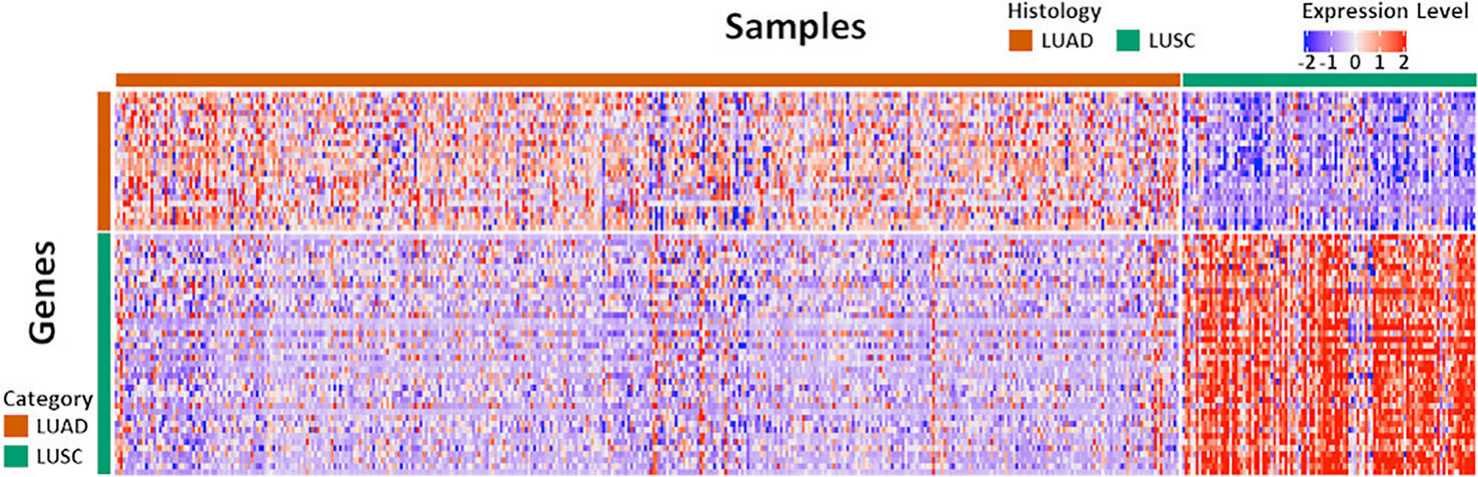
The expression pattern of the 67 diagnostic genes in the Exploration Cohort. The Exploration Cohort was composed of 490 samples collected from LUAD (n = 384) and LUSC (n = 106) tumors. The 67-gene diagnostic panel was composed of 27 LUAD-specific and 40 LUSC-specific genes. The gene expression pattern was derived from the normalized microarray-based transcriptomics data and scaled for visualization.

The original NanoString 67-gene panel data as well as the individually normalized gene profile of the Exploring cohort and FNA cohort were visualized in **Figure 2** and **Supplement Figure S1**, respectively. The panel was composed of 63 candidate diagnostic genes and, for internal normalization purpose, 4 housekeeping genes. The candidate diagnostic genes demonstrated strong differential expression pattern in the LUAD vs. LUSC samples. Specifically, the 23 LUAD-specific genes were highly expressed in LUAD samples, and vice versa for the 40 LUSC-specific genes. To reach a robust model, a large-scale resampling for cross-validation was performed over the full range of elastic net mixing factor *α*. Through 1,695,000 training-and-testing rounds, the best performance of an accuracy of 0.902 ± 0.053 was achieved at *α* = 0.5 with a model complexity of 15 predictive genes **(Supplement: Feature Selection** and **Figure S2** and **Figure S3**). The boxplots of key model features such as the optimal *α*, *λ*, accuracy, and the model complexity during the first 100 resampling were shown in **Supplement Figure S4**. The convergence of model training during resampling was shown in **Supplement Figure S5**.

### The Molecular Subtyping Model

**Figure 3:**
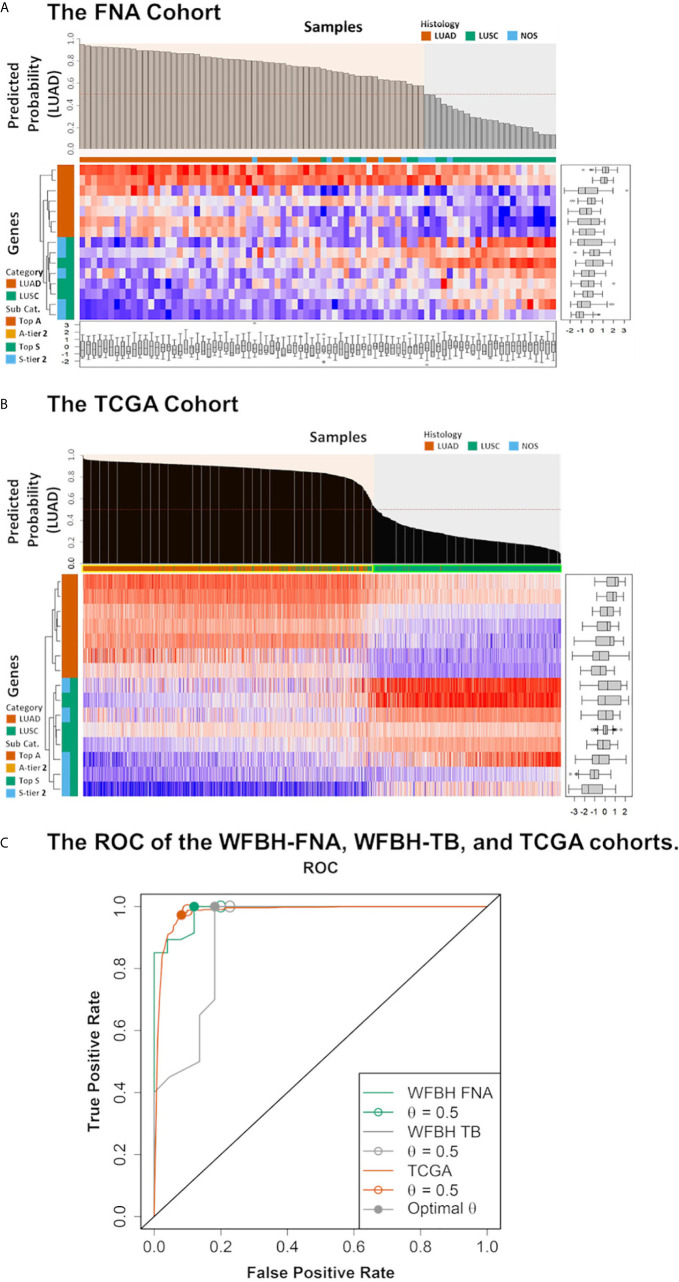
Model performance. The predicted probabilities of subtype LUAD for **(A)** each FNA samples and **(B)** each TCGA samples was visualized in the bar plot. Samples classified as LUAD (p ≥ 0.5) or LUSC (p < 0.5). Signature genes used in the molecular subtyping model were listed according to the absolute values of their logistic regression coefficients. The categories of the genes were color coded with respect to the associated subtypes (vermilion for LUAD and bluish green for LUSC). **(C)** The receiver operating characteristic curves (ROCs) of the model performance on the WFBH FNA, the WFBH tissue bank, and the TCGA cohorts. The corresponding c-statistics (AUC, area under the ROC curve) were 0.986, 0.911, and 0.982, respectively. When the probability threshold is set at *θ* = 0.5, model accuracies are 0.931, 0.881, and 0.945 for the WFBH FNA, the WFBH tissue bank, and the TCGA cohorts, respectively (marked as open circles). The optimal accuracies are reached when optimal *θ* levels are used, which are: 0.958 at *θ* = 0.60 for the WFBH FNA cohort, 0.905 at *θ* = 0.59 for the WFBH tissue bank cohort, and 0.946 at *θ* = 0.68 for the TCGA cohort (solid gray circles).

The final 15 signature genes as well as corresponding coefficients were listed in **Table 2** and **Supplement Table S2**. The performance of the model on FNA cohort was demonstrated in **Figure 3** and listed in **Supplement Table S3**.

**Table 2:** Genes and coefficients of the molecular subtyping model.

Category	Gene	coefficient
LUSC-related	TP63	-0.58872
LUSC-related	KRT14	-0.39198
LUSC-related	ANXA8L2	-0.27091
LUSC-related	KRT5	-0.25936
LUSC-related	SERPINB13	-0.10086
LUSC-related	SNAI2	-0.048
LUSC-related	KRT6A	-0.0221
LUSC-related	PKP1	-0.00488
	(Intercept)	0.27297
LUAD-related	SPINK1	0.00302
LUAD-related	CD55	0.00562
LUAD-related	NKX2-1	0.02709
LUAD-related	MUC1	0.13468
LUAD-related	GPR116	0.13792
LUAD-related	PNMA2	0.15816
LUAD-related	TMC5	0.31305

Signature genes used in the molecular subtyping model were listed according to the absolute values of their logistic regression coefficients. The categories of the genes were color coded with respect to the associated subtypes (vermilion for LUAD and bluish green for LUSC). Genes are sorted according to significance (absolute values of the corresponding coefficients, with positive values favoring LUAD and negative values favoring LUSC subtypes).

The intensive resampling was crucial since the sample size was relatively small (n = 72) comparing with the candidate gene set (m = 63). As shown in **Supplement Figure S4**, significant variations were observed for the FNA dataset, comparing with the much more stable results for The Cancer Genome Atlas (TCGA) dataset (n = 1,016) (**Supplement Figure S5**). The convergence analysis (**Supplement Figure S4**) showed that, to confidently estimate the model complexity and the elastic net mixing factor, at least 1,000 rounds of resampling was required. Resampling approach also provided a realistic estimation of the model performance (**Supplement Figure S5**). The model performance on internal tissue bank dataset (accuracy of 89.3%) and on external TCGA dataset (accuracy of 94.5%) were consistent with the predicted performance (0.903 ± 0.053).

### Model Validation

The model was validated internally with the 67-gene panel transcriptomics generated on the NanoString platform with frozen tumor LUAD and LUSC tissues, and externally with the RNA-Seq transcriptomics from TCGA LUAD and LUSC dataset. The performance on the WFBH tissue bank cohort and on the TCGA cohort were shown in **Supplement Tables S4**, **S5**, and **Figure 3**, respectively. The c-statistics of the model on the WFBH-FNA, WFBH-TB, and the TCGA cohorts were 0.986, 0.911, and 0.982, respective, with the corresponding Receiver Operating Characteristic (ROC) curves shown in **Figure 3C**.

Control of overfitting is a major challenge in molecular subtyping, since comparing with the large size of candidate biomarkers made possible by modern high-throughput or multiplex technologies. The clinical cohorts are usually small. We have significantly reduced the candidate biomarkers from microarray data to 63 genes. Such a focused gene panel still raise overfitting concern for our clinical cohort. With elastic net regularization and intensive resampling, we reduced the model complexity (gene size = 15) and achieved an events-per-variable (EPV) level of about 5. Such EPV level was around the minimum statistically acceptable (36) ratio for model development. Therefore, the validation independent dataset was necessary. The TCGA cohort validation confirmed that the established molecular subtyping model was not overfitted.

### Choosing Thresholds for Confident Subtyping in Clinical Implementation

In clinical practice, it is important to identify which cases can be confidently subtyped without pathologists’ review. Subtype-specific thresholds, *θ_LUAD_* and *θ_LUSC_*, can be used to reach required confidence. Samples falling between these thresholds need to be examined by pathologists. As shown in **Supplement Figure S6**, 94.7% of clinical samples can be confidently subtyped with 95% specificity when using *θ_LUAD_* = 0.84 and *θ_LUSC_* = 0.74. About 87.5% clinical samples can be subtyped at a specificity of 97% if thresholds *θ_LUAD_* = 0.89 and *θ_LUSC_* = 0.66 are used.

### Comparison With Current Subtyping Approaches

The classification model demonstrated high accuracy when compared to standard NSCLC clinical subtyping. The TCGA LUAD and LUSC cohort study model outperformed the histopathological image-based artificial intelligence approach using deep convolutional neural network (37), with an AUC of 98.2% vs. 97%. On clinical samples, a subtyping specificity of 97% is at the high-end of current pathological diagnosis utilizing immunohistochemical markers (38). The performance analysis showed that only about 12.5% clinical samples are below this specificity and thus need to be further reviewed by pathologists. Implementing our subtyping model will significantly relieve the burden of pathologists.

### NOS Case Profiling

**Figure 4:**
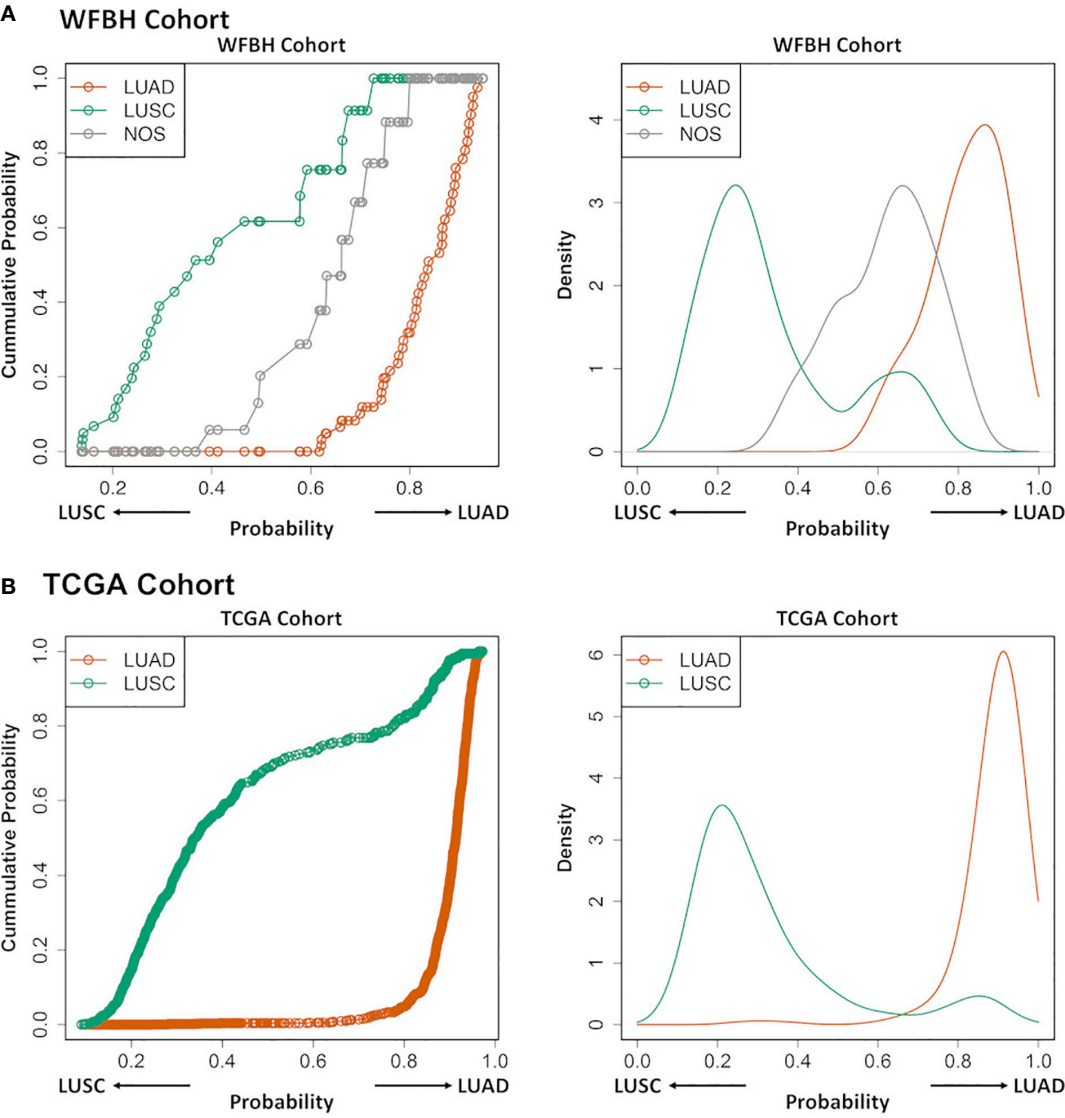
Profiling LUAD and LUSC subtypes and NOS cases. The cumulative probability distribution curves (left) and the estimated probability density distribution curves (right) for the prediction results of the LUAD, LUSC, and NOS cases in WFBH **(A)** and TCGA **(B)** cohorts, respectively. Each circle in the left panels represents a sample.

The NOS cases in the WFBH-TB cohort were examined using the developed molecular subtyping model and the subtypes of these cases predicted (**Figure 3**). Further analysis demonstrated unique subtyping probability distribution of NOS cases (the gray cumulative density function curve and the gray probability density function peak in the left and right panel of **Figure 4A**) comparing with typical LUAD and LUSC cases (Kolmogoro-Smirnov test p-values are 2×10^–4^ for both NOS vs. LUAD and NOS vs. LUSC). In contrast, there was no similar NOS-like probability density function peak in TCGA cohort (right panel in **Figure 4B**), in which the NOS cases were excluded. The profiling results suggested that the NOS cases at WFBH cohort might be molecularly distinct from typical LUAD and LUSC subtypes.

### Clinical Relevance

Our work proved the concept that *a Comprehensive, Computable NanoString Diagnostic gene panel* (C2Dx) is a promising tool to conserve diagnostic tissue specimens that require molecular pathological diagnosis when limited small volume tumor specimens are available. The C2Dx platform was developed from underlying classification models for molecular diagnosis, the NanoString gene panel to reliably generate targeted genomics data, and the clinical and experimental protocols for obtaining FNA specimens and generating NanoString genomics data. C2Dx platform demonstrates high potential in addressing the challenges in molecular diagnosis on FNA samples. The current advance of clinical practice raises a challenge to traditional pathology approaches: the gap between the dramatically reduced sample volume when less invasive approaches such as FNA are used and the fast-increasing needs of molecular diagnosis required by modern therapies such as immune therapies, targeted therapies, and, of course, chemotherapies. NanoString data generated from the clinical FNA samples can address such clinical need. Our approach provides a reliable clinical solution to this emerging clinical need.

We chose to use the LUAD vs. LUSC subtyping to prove the concept of C2Dx. First, the task is representatively complex for typical molecular diagnosis tasks, and requires a large number of genes used in a multivariate model. It is both experimentally and mathematically more complex than the standard PD-1/PD-L1 test which only requires a single or few genes. It is representative for other molecular diagnostic needs by using metagenes and gene signatures. Second, this task is suitable for examining the performance of the C2Dx platform. It has been well-established clinically, pathologically, and molecularly. Finally, subtyping LUAD vs. LUSC cases is still an essential pathological task in clinical practice and impacts treatment decisions.

Beyond the use of the C2Dx platform for fresh FNA samples, the computable subtyping model can be applied for other clinical scenarios. Scenario 1: centralized C2Dx laboratory services, as many clinical sites rely on such external resources for genomics profiling tasks. Scenario 2: retrospective calibration and molecular diagnosis for frozen samples stored in tissue banks at low cost and low demands of sample volume. Our work demonstrated that reliable molecular diagnoses can be achieved for frozen samples and thus both scenarios can be archived.

Finally, our subtyping model allows linking the emerging clinical FNA samples to previous patient cohorts for integrative analysis. For example, samples collected at WFBH are comparable to TCGA samples using the subtyping probability scores calculated from the logistic subtyping model on the nanoString 19-gene panel data and the TCGA’s RNA-Seq transcriptomic data. Such mapping provides comprehensive, multi-omics molecular contexts to individual clinical cases, and enables translations of knowledge from public biomedical big data to support precision oncology for individual patients.

### Limitations and Future Directions

The main goal of this work is to use a well-established molecular subtyping task (distinguishing LUSC from LUAD cases among NSCLC patients) to prove the concept of a crucial step toward a comprehensive, computable gene panel for molecular diagnosis – robust molecular modeling for bench- -to-bedside knowledge translation. To be implemented in real-world clinical settings, the model needs to be expanded to distinguish small-cell lung cancer cases. Another major future direction will be the incorporation of biomarkers representing the pathological and prognostic knowledge from different domains including genomic abnormalities such as EGFR and BRAF mutations, molecular subtypes such as LUSC and LUAD, immunotherapy markers such as PD-1/PD-L1 expression, and other transcriptomic and genomic signatures for precision oncology.

## Conclusions

Our work using FNA-based nanoString gene panel for robust molecular subtyping of NSCLC patients dramatically can reduce the demand of diagnostic samples for subtyping. It can avoid the complications and risks of re-biopsy when specimens are limited. It allows for more tissues to support molecular diagnoses for emerging targeted and immune therapies, and release pathologists from subtyping burden. It may also be used in the research setting, when utilizing small volume “left over” sampling. This translational modeling work is a crucial step toward comprehensive, computable molecular diagnosis devices and potentially revolutionizes current NSCLC diagnosis procedures for precision oncology

## Data Availability Statement 

The original contributions presented in the study are included in the article/**Supplementary Material**. Further inquiries can be directed to the corresponding author. The 19-gene molecular subtyping model was also available as an R package at http://github.com/Su-informatics-lab/FNASubtype, with sample R code demonstrated in the supplements.

## Ethics Statement

The study was approved by the Institutional Review Board of the Wake Forest Baptist Medical Center. The patients/participants provided their written informed consent to participate in this study.

## Author Contributions

JS, LM, and JR prepared the manuscript. LM and JR designed the experiments. JS and LH analyzed the data. RB developed the github distribution of the tool. GP, JC, CB, TD, LC, IP, JH, HC, and JP contributed to sample collection and data generation. JS, LH, BPr, MC, LM, and JR participated the development of the manuscript. All authors contributed to the article and approved the submitted version.

## Funding

The work is partially supported by the Cancer Center Support Grant from the National Cancer Institute to the Comprehensive Cancer Center of Wake Forest Baptist Medical Center (P30 CA012197). The work is also partially supported by the Indiana University Precision Health Initiative (funded to JS). The content is solely the responsibility of the authors and does not necessarily represent the official views of the National Institutes of Health.

## Conflict of Interest

The authors declare that the research was conducted in the absence of any commercial or financial relationships that could be construed as a potential conflict of interest.

## References

[1] Dy GK Nesline MK Papanicolau-Sengos A DePietro P LeVea CM Early A. Treatment recommendations to cancer patients in the context of FDA guidance for next generation sequencing. BMC Med Inform Decis Mak (2019) 19:14. 10.1186/s12911-019-0743-x 30658646 PMC6339275

[2] Yatabe Y Dacic S Borczuk AC Warth A Russell PA Lantuejoul S. Best Practices Recommendations for Diagnostic Immunohistochemistry in Lung Cancer. J Thorac Oncol (2019) 14:377–407. 10.1016/j.jtho.2018.12.005 30572031 PMC6422775

[3] Ettinger DS Aisner DL Wood DE Akerley W Bauman J Chang JY. NCCN Guidelines Insights: Non-Small Cell Lung Cancer, Version 5.2018. J Natl Compr Canc Netw (2018) 16:807–21. 10.6004/jnccn.2018.0062 30006423

[4] Travis WD Brambilla E Burke AP Marx A Nicholson AG. Introduction to The 2015 World Health Organization Classification of Tumors of the Lung, Pleura, Thymus, and Heart. J Thorac Oncol (2015) 10:1240–2. 10.1097/JTO.0000000000000663 26291007

[5] Fassina A Cappellesso R Simonato F Lanza C Marzari A Fassan M. Fine needle aspiration of non-small cell lung cancer: current state and future perspective. Cytopathology (2012) 23:213–9. 10.1111/j.1365-2303.2012.01005.x 22805511

[6] Micames CG McCrory DC Pavey DA Jowell PS Gress FG. Endoscopic ultrasound-guided fine-needle aspiration for non-small cell lung cancer staging: A systematic review and metaanalysis. Chest (2007) 131:539–48. 10.1378/chest.06-1437 17296659

[7] Tutar N Yurci A Gunes I Gulmez I Gursoy S Onal O. The role of endobronchial and endoscopic ultrasound guided fine needle aspiration for mediastinal nodal staging of non-small-cell lung cancer. Tuberk Toraks (2018) 66:85–92. 10.5578/tt.66866 30246650

[8] Brainard J Farver C. The diagnosis of non-small cell lung cancer in the molecular era. Mod Pathol (2019) 32:16–26. 10.1038/s41379-018-0156-x 30600321

[9] National Research Council (US) Committee on A Framework for Developing a New Taxonomy of Disease. Toward Precision Medicine: Building a Knowledge Network for Biomedical Research and a New Taxonomy of Disease. Washington (DC): National Academies Press (US) (2011). 22536618

[10] Prasad V Fojo T Brada M. Precision oncology: origins, optimism, and potential. Lancet Oncol (2016) 17:e81–6. 10.1016/S1470-2045(15)00620-8 26868357

[11] Brown NA Elenitoba-Johnson KSJ. Enabling Precision Oncology Through Precision Diagnostics. Annu Rev Pathol (2020) 15:97–121. 10.1146/annurev-pathmechdis-012418-012735 31977297

[12] Young G Wang K He J Otto G Hawryluk M Zwirco Z. Clinical next-generation sequencing successfully applied to fine-needle aspirations of pulmonary and pancreatic neoplasms. Cancer Cytopathol (2013) 121:688–94. 10.1002/cncy.21338 23893923

[13] Beltran H Yelensky R Frampton GM Park K Downing SR MacDonald TY. Targeted next-generation sequencing of advanced prostate cancer identifies potential therapeutic targets and disease heterogeneity. Eur Urol (2013) 63:920–6. 10.1016/j.eururo.2012.08.053 PMC3615043 22981675

[14] Elkhouly E Zhou L Kurtis B Wang L Israyelyan A. Real-world experience in advanced NSCLC using FDA approved NGS CDx. J Clin Oncol (2019) 37:e14602–2. 10.1200/JCO.2019.37.15_suppl.e14602

[15] Yu TM Morrison C Gold EJ Tradonsky A Layton AJ. Multiple Biomarker Testing Tissue Consumption and Completion Rates With Single-gene Tests and Investigational Use of Oncomine Dx Target Test for Advanced Non-Small-cell Lung Cancer: A Single-center Analysis. Clin Lung Cancer (2019) 20:20–29 e8. 10.1016/j.cllc.2018.08.010 30243889

[16] De Rubis G Rajeev Krishnan S Bebawy M. Liquid Biopsies in Cancer Diagnosis, Monitoring, and Prognosis. Trends Pharmacol Sci (2019) 40:172–86. 10.1016/j.tips.2019.01.006 30736982

[17] Bracht JWP Mayo-de-Las-Casas C Berenguer J Karachaliou N Rosell R. The Present and Future of Liquid Biopsies in Non-Small Cell Lung Cancer: Combining Four Biosources for Diagnosis, Prognosis, Prediction, and Disease Monitoring. Curr Oncol Rep (2018) 20:70. 10.1007/s11912-018-0720-z 30030656

[18] Geiss GK Bumgarner RE Birditt B Dahl T Dowidar N Dunaway DL. Direct multiplexed measurement of gene expression with color-coded probe pairs. Nat Biotechnol (2008) 26:317–25. 10.1038/nbt1385 18278033

[19] Tsang HF Xue VW Koh SP Chiu YM Ng LP Wong SC. NanoString, a novel digital color-coded barcode technology: current and future applications in molecular diagnostics. Expert Rev Mol Diagn (2017) 17:95–103. 10.1080/14737159.2017.1268533 27917695

[20] Eastel JM Lam KW Lee NL Lok WY Tsang AHF Pei XM. Application of NanoString technologies in companion diagnostic development. Expert Rev Mol Diagn (2019) 19:591–8. 10.1080/14737159.2019.1623672 31164012

[21] Ali G Bruno R Savino M Giannini R Pelliccioni S Menghi M. Analysis of Fusion Genes by NanoString System: A Role in Lung Cytology? Arch Pathol Lab Med (2018) 142:480–9. 10.5858/arpa.2017-0135-RA 29372843

[22] Veldman-Jones MH Lai Z Wappett M Harbron CG Barrett JC Harrington EA. Reproducible, Quantitative, and Flexible Molecular Subtyping of Clinical DLBCL Samples Using the NanoString nCounter System. Clin Cancer Res (2015) 21:2367–78. 10.1158/1078-0432.CCR-14-0357 25301847

[23] Dotson T Bellinger C Su J Hansen K Parks GE Cappellari JO. Feasibility of lung cancer RNA acquisition from a single transbronchial or transthoracic needle pass (FASTT trial). Lung Cancer (2019) 127:6–11. 10.1016/j.lungcan.2018.11.023 30642553 PMC6386467

[24] Su J Jin G Votanopoulos KI Craddock L Shen P Chou JW. Prognostic Molecular Classification of Appendiceal Mucinous Neoplasms Treated with Cytoreductive Surgery and Hyperthermic Intraperitoneal Chemotherapy. Ann Surg Oncol (2020) 27(5):1439–47. 10.1245/s10434-020-08210-5 PMC7147286 31980985

[25] Broet P Camilleri-Broet S Zhang S Alifano M Bangarusamy D Battistella M. Prediction of clinical outcome in multiple lung cancer cohorts by integrative genomics: implications for chemotherapy selection. Cancer Res (2009) 69:1055–62. 10.1158/0008-5472.CAN-08-1116 19176396

[26] Raponi M Zhang Y Yu J Chen G Lee G Taylor JM. Gene expression signatures for predicting prognosis of squamous cell and adenocarcinomas of the lung. Cancer Res (2006) 66:7466–72. 10.1158/0008-5472.CAN-06-1191 16885343

[27] Bild AH Yao G Chang JT Wang Q Potti A Chasse D. Oncogenic pathway signatures in human cancers as a guide to targeted therapies. Nature (2006) 439:353–7. 10.1038/nature04296 16273092

[28] A. Director’s Challenge Consortium for the Molecular Classification of Lung Shedden K Taylor JM Enkemann SA Tsao MS Yeatman TJ. Gene expression-based survival prediction in lung adenocarcinoma: a multi-site, blinded validation study. Nat Med (2008) 14:822–7. 10.1038/nm.1790 PMC2667337 18641660

[29] Silva TC Colaprico A Olsen C D’Angelo F Bontempi G Ceccarelli M. TCGA Workflow: Analyze cancer genomics and epigenomics data using Bioconductor packages. F1000Res (2016) 5:1542. 10.12688/f1000research.8923.1 28232861 PMC5302158

[30] Zou H Hastie T. Regularization and variable selection via the elastic net. J R Stat Soc Ser B (Stat Methodol) (2005) 67:301–20. 10.1111/j.1467-9868.2005.00503.x

[31] Friedman J Hastie T Tibshirani R. Regularization Paths for Generalized Linear Models via Coordinate Descent. J Stat Softw (2010) 33:1–22. 10.18637/jss.v033.i01 20808728 PMC2929880

[32] Kuhn M. Caret package. J Stat Softw (2008) 28:1–26. 10.18637/jss.v028.i05 27774042

[33] Spackman KA. Signal detection theory: Valuable tools for evaluating inductive learning. In: Proceedings of the sixth international workshop on Machine learning. Burlington, MA: Morgan Kaufmann Publishers Inc. (1989). p. 160–3.

[34] Hanley JA McNeil BJ. A method of comparing the areas under receiver operating characteristic curves derived from the same cases. Radiology (1983) 148:839–43. 10.1148/radiology.148.3.6878708 6878708

[35] Fawcett T. An introduction to ROC analysis. Pattern Recognit Lett (2006) 27:861–74. 10.1016/j.patrec.2005.10.010

[36] Vittinghoff E McCulloch CE. Relaxing the rule of ten events per variable in logistic and Cox regression. Am J Epidemiol (2007) 165:710–8. 10.1093/aje/kwk052 17182981

[37] Coudray N Ocampo PS Sakellaropoulos T Narula N Snuderl M Fenyo D. Classification and mutation prediction from non-small cell lung cancer histopathology images using deep learning. Nat Med (2018) 24:1559–67. 10.1038/s41591-018-0177-5 PMC9847512 30224757

[38] Ebrahimi M Auger M Jung S Fraser RS. Diagnostic concordance of non-small cell lung carcinoma subtypes between biopsy and cytology specimens obtained during the same procedure. Cancer Cytopathol (2016) 124:737–43. 10.1002/cncy.21739 27172103

