# Supplementary material for: Comprehensive and Computable Molecular Diagnostic Panel (C2Dx) From Small Volume Specimens for Precision Oncology: Molecular Subtyping of Non-Small Cell Lung Cancer From Fine Needle Aspirates
Source: Front Oncol. 2021 Apr 16;11:584896. doi: 10.3389/fonc.2021.584896 (PMC8085404; doi:10.3389/fonc.2021.584896)
Supplement: Supplementary file 1 [file DataSheet_1.docx]

Supplementary Material

Title: Comprehensive and Computable Molecular Diagnostic Panel (C2Dx) from Small Volume Specimens for Precise Oncology: Molecular Subtyping of Non-Small Cell Lung Cancer from Fine Needle Aspirates

**Authors:**

Jing Su^1,2,3^, Lynn S. Huang^3^, Qianqian Song^1^, Ryan Barnard^3^, Graham Parks^4^, James Capillari^4^, Christina Bellinger^5^, Travis Dotson^5^, Lou Craddock^1^., IB. Prakash^5^, Jonathan Hovda^6^, Hollins Clark^7^,W. Jeffrey Petty^8^, Boris Pasche^1^, Michael D. Chan^6^, Lance D. Miller^1^, Jimmy Ruiz^8, 9^

^1^Department of Cancer Biology, Wake Forest School of Medicine, Winston-Salem, NC

^2^Department of Biostatistics, Indiana University School of Medicine, Indianapolis, IN

^3^Department of Biostatistics and Data Science, Wake Forest School of Medicine, Winston-Salem, NC

^4^Department of Pathology, Wake Forest School of Medicine, Winston-Salem, NC

^5^Department of Medicine (Pulmonology and Critical Care), Wake Forest School of Medicine, Winston-Salem, NC

^6^Department of Radiation Oncology, Wake Forest School of Medicine, Winston-Salem, NC

^7^Department of Radiology, Wake Forest School of Medicine, Winston-Salem, NC

^8^Department of Medicine (Hematology & Oncology), Wake Forest School of Medicine, Winston-Salem, NC

^9^W.G. (Bill) Hefner Veteran Administration Medical Center, Cancer Center, Salisbury, NC

Corresponding author:

Jimmy Ruiz

jruiz@wakehealth.edu

**Table of Contents**

[1. Construction of the Microarray Meta-Cohort 3](#_Toc45891676)

[2. Construction of NanoString Gene Panel 3](#_Toc45891677)

[3. Data Preprocessing 4](#_Toc45891678)

[4. Model Selection 5](#_Toc45891679)

[Figure S1: Gene expression profile of the individually normalized NanoString transcriptomics for FNA samples. 6](#_Toc45891680)

[Figure S2: Diagram for elastic net model selection. 7](#_Toc45891681)

[Figure S3: Cross validation for elastic net regularization 8](#_Toc45891682)

[Figure S4: Convergence of key features during resampling for the FNA dataset 9](#_Toc45891683)

[Figure S5: Convergence of model accuracy during resampling for the FNA dataset 11](#_Toc45891684)

[Figure S6: Model thresholds for confident subtyping in clinical implementation. 12](#_Toc45891685)

[Table S1. Population characteristics of the Exploring meta-cohort. 13](#_Toc45891686)

[Table S2: Signature genes and coefficients of the molecular subtyping model 14](#_Toc45891687)

[Table S3: Performance on FNA cohort 15](#_Toc45891688)

[Table S4: Performance on WFBH tissue bank cohort 16](#_Toc45891689)

[Table S5: Performance on TCGA cohort 17](#_Toc45891690)

[Table S6: Probes on the 67-gene panel 18](#_Toc45891691)

[Sample Code 19](#_Toc45891692)

[References 20](#_Toc45891693)

# Construction of the Microarray Meta-Cohort

To construct and validate a gene-expression based outcome predictor for early stage NSCLC with maximal statistical power, we compiled a multi-institutional microarray “meta-cohort” comprising of 490 Stage I NSCLC cases, where each was profiled on Affymetrix GeneChips from surgically-resected, fresh frozen tumor specimens. The cohort was comprised of 4 independent lung cancer cohorts (Paris[1], Michigan[2], Duke[3] and the Director’s Challenge[4] assembled from 7 institutions: Univ. of Michigan Cancer Center, Moffitt Cancer Center, Memorial Sloan-Kettering Cancer Center, Univ. of Minnesota VA/CALGB, Univ. Health Network Toronto, and the NCIC Clinical Trials Group) whose clinical characteristics are presented in **Table S1**. The Microarray Meta-Cohort cases were derived from 9 different medical centers in North America and Europe, and together totaled 757 primary NSCLC cases ranging from stage I-IV. Data sets and associated clinical annotations were downloaded from the Gene Expression Omnibus (http://www.ncbi.nlm.nih.gov/geo; accession numbers: GSE10445[1], GSE4573[2], and GSE3141[3]), and the NCI’s *caArray* microarray database (https://array.nci.nih.gov/caarray/home.action; accession number jacob-00182). All tumor samples were analyzed from surgically resected frozen tissue and profiled on the Affymetrix U133A or U133 PLUS 2.0 GeneChip, according to standard Affymetrix protocols as we previously reported [5]. For all cases, raw data (CEL files) were quantile-normalized within cohorts by computing Robust Multichip Average (RMA) expression values. Batch effects between cohorts were estimated by mixed model ANOVA and corrected using the Partek Genomics Suite Batch Remover program. The complete matrix of RMA-normalized, batch-corrected microarray expression data and corresponding clinico-pathological tumor annotations can be found in **Supplemental Datafiles 1 and 2**, respectively.

# Construction of NanoString Gene Panel

The transcriptomic data and diagnostic results of subtypes (LUAD or LUSC) of the Microarray Meta-Cohort were used to identify candidate, subtype-associated genes suitable for the NanoString gene panel. As previously reported[6], candidate genes were comprehensively screened according to a battery of scientific, operational, and pragmatic criteria including the biological and statistical significance associated with the two subtypes, the capability to classify LUAD and LUSC subtypes, the overall expression level, the robustness across different screening approaches, the availability and specificity of the corresponding NanoString probes, and the potential cost which is crucial for translating the gene panel to affordable clinical diagnostic devices. Cases were first evenly assigned to *training* and *test* sets for classifier construction and validation (n=245 in each set). Candidate genes were selected using the training set and evaluated using the testing set. A comprehensive approach was designed and used to robustly screen genomic classifiers associated with these two subtypes. This approach was featured by the control of sensitivity to algorithm-specific classifiers. To reduce bias due to the classification algorithms, we used concordance analysis across four classification algorithms including Diagonal Linear Discriminant Analysis (DLDA)[7], Support Vector Machines (SVM)[8, 9], Nearest Centroids (NC)[10, 11], and Bayesian Compound Covariate Prediction (BCCP)[12] using BRB-Array Tools[13, 14]. A standardized feature selection strategy was employed based on three threshold significance levels: α=0.01, α=0.001, or α=0.0001. At each significance level, genes were selected for model inclusion using leave-one-out cross-validation, respectively. Thus, we examined a total of 12 different classification models (4 algorithms and 3 α levels). Finally, 63 subtype-specific genes and 4 housekeeping genes (for normalization) were selected.

# Data Preprocessing

Expression data, either in the raw Reporter Code Count for NanoString transcriptomics or in the FPKM-UQ in the TCGA RNA-Seq transcriptomics, were normalized for each individual sample using the 4 housekeeping genes, which were: ACTB (β actin), RPL39 (ribosomal protein L39), TPT1 (tumor protein, translationally-controlled 1) and EEF1A1 (eukaryotic translation elongation factor 1 alpha 1).

**NanoString data.** The raw Reporter Code Count result $C_{i,j}$ for sample $i$ and gene $j$ was first normalized against the gene expression of the housekeeping genes of the same sample:

$$\hat{C}_{i,j}=\frac{\log_{2} C_{i,j}}{\frac{\left( \log_{2} C_{i,ATCB}+\log_{2} C_{i,RPL39}+\log_{2} C_{i,TPT1}+\log_{2} C_{i,EEF1A1} \right)}{4}}$$

Then the normalized expression data of a sample was further standardized by z-transformation:

$$g_{i,j}=\frac{\hat{C}_{i,j}-\text{mean}\left( \hat{C}_{i,\cdot} \right)}{\text{sd}\left( \hat{C}_{i,\cdot} \right)}$$

**TCGA data.** The TCGA FPKM-UQ result ${FPKM}_{i,j}$ of the 67 genes for sample $i$ and gene $j$ was first normalized against the gene expression of the housekeeping genes of the same sample:

$$\hat{FPKM}_{i,j}=\frac{\log_{2} {FPKM}_{i,j}}{\frac{\left( \log_{2} {FPKM}_{i,ATCB}+\log_{2} {FPKM}_{i,RPL39}+\log_{2} {FPKM}_{i,TPT1}+\log_{2} {FPKM}_{i,EEF1A1} \right)}{4}}$$

Then the normalized expression data of a sample was further standardized by z-transformation:

$$g_{i,j}=\frac{\hat{FPKM}_{i,j}-\text{mean}\left( \hat{FPKM}_{i,\cdot} \right)}{\text{sd}\left( \hat{FPKM}_{i,\cdot} \right)}$$

As an example, the normalized data for the FNA cohort was shown in Figure S1.

# Model Selection

Elastic net regularization with 3-fold cross validation and 10,000-time resampling was used for feature selection (Figure S2). The elastic net mixing factor *α* was screened from 0 to 1 at 0.1 interval, with the elastic net penalty factor *λ* screened within a range adaptively determined according to the model performance. Totally 565 elastic net parameter combinations (Figure S3) were examined (11 levels for *α* and 55 levels for *λ*). For each combination, 30,000 cross validation were performed. Through 16,950,000 training and testing, the best performance of an accuracy of 0.902±0.053 was achieved at *α* = 0.5, *λ* = 0.177, with a model complexity of 15 predictive genes. For the given modest sample size (n = 72) and the large gene size (m = 63), large scale resampling was crucial to robustly determine the model complexity (that is, how many gene should be included in the model). The convergence of the overall model training during resampling was shown in Figure S4. The optimal values of key parameters *α* and *λ* were cumulatively identified during the resampling. For example, the first 10 resampling results suggested that the optimal values were *α* = 0.8 and *λ* = 0.177, which the first 90 resampling results suggested *α* = 0.1 and *λ* = 0.406. Our results suggested that the estimation of the key parameters in the elastic net model became stable after 1,000 resampling. Meanwhile, the cumulative estimation of model accuracy became stable after 8,000 resampling (Figure S5).

Figure S1: Gene expression profile of the individually normalized NanoString transcriptomics for FNA samples.


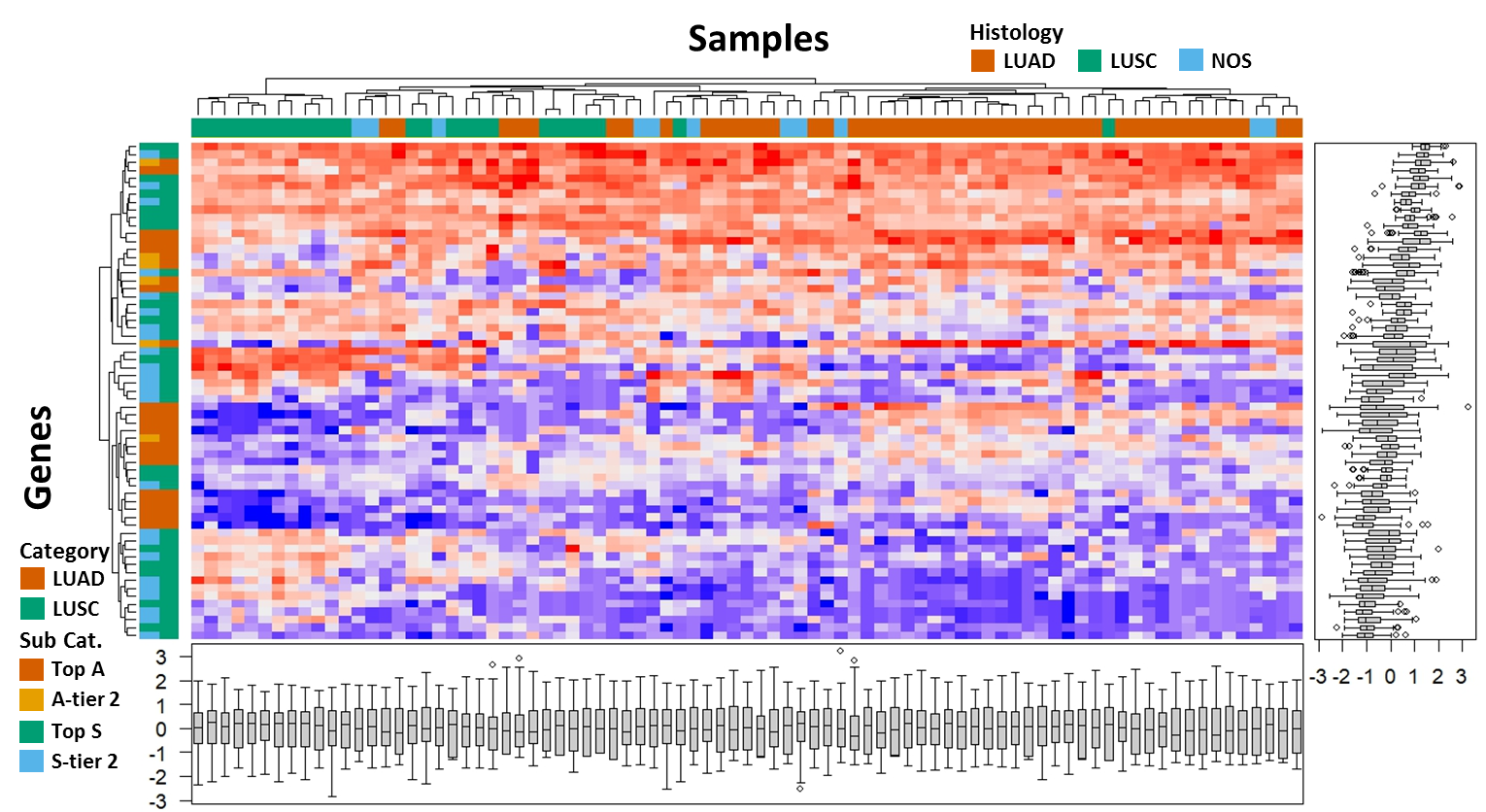


Figure S2: Diagram for elastic net model selection.


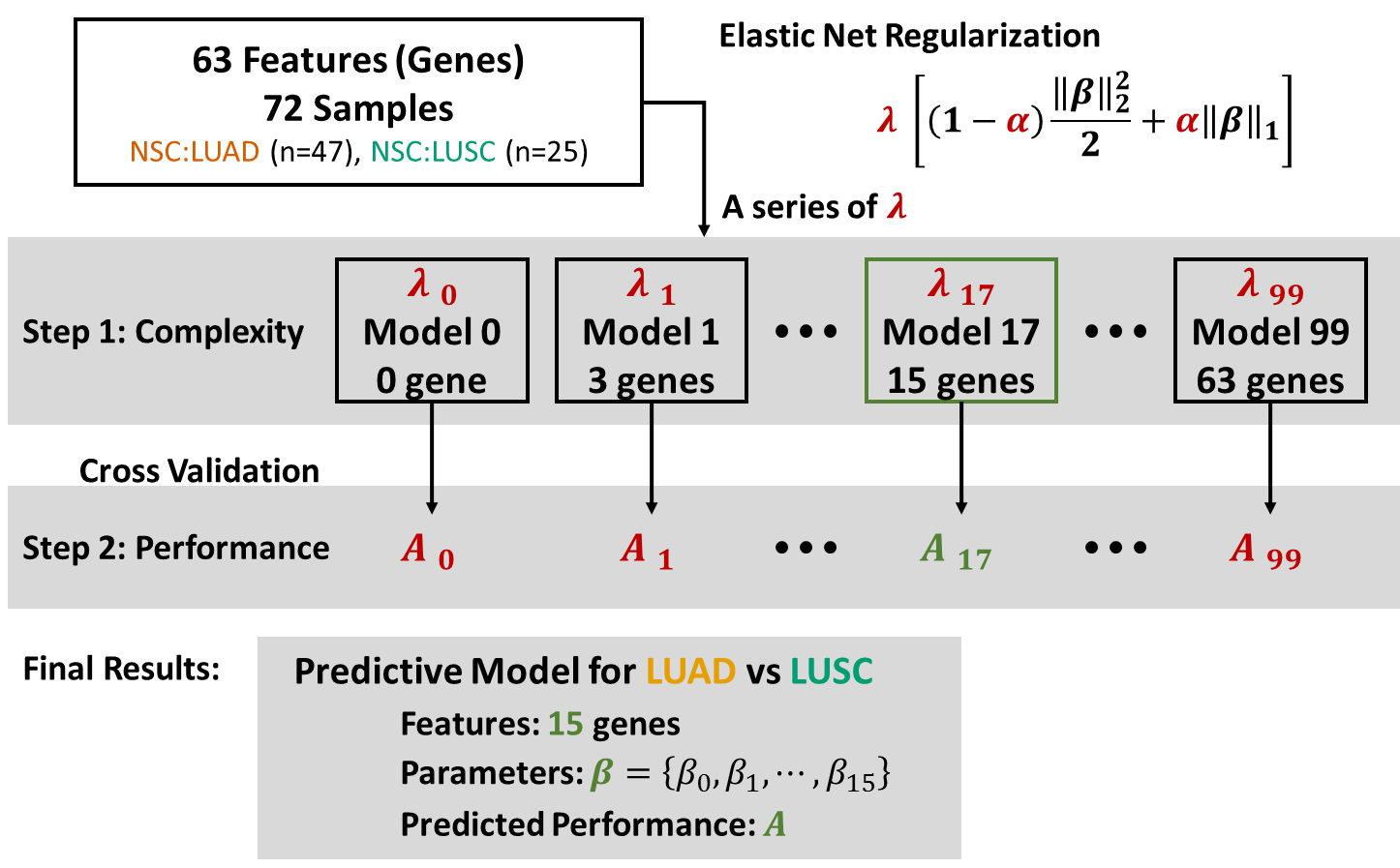


Figure S3: Cross validation for elastic net regularization

Each line represents a specific elastic net mixing percentage *α*, ranging from 0 to 1. The best performance of 0.902 achieved at $\alpha=0.5$ and $\lambda=$0.177 (green curve and filled green circle).

Figure S4: Convergence of key features during resampling for the FNA dataset

(A) The optimal *α* cumulatively identified during the first 1,000 resampling.


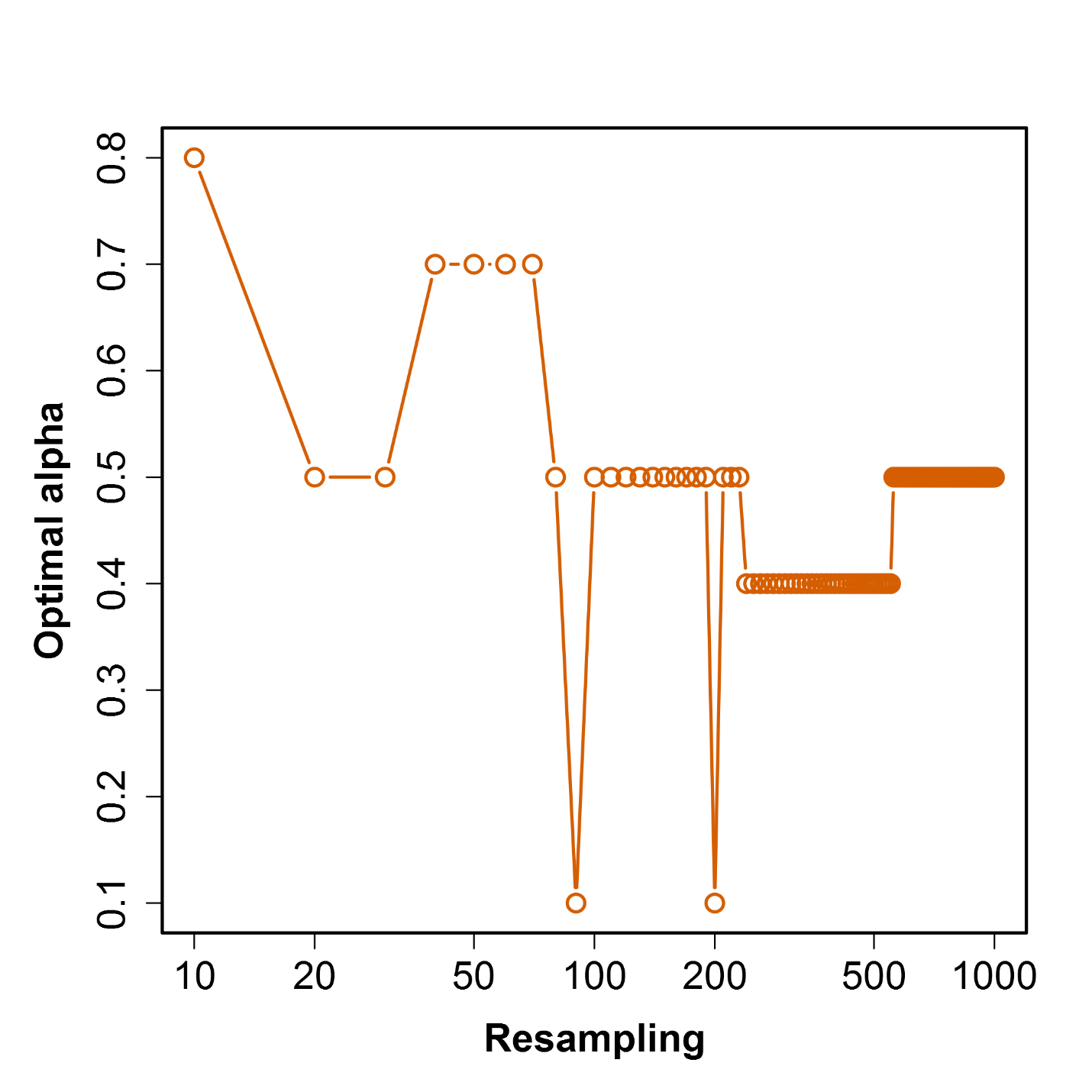


(B) The optimal *λ* cumulatively identified during the first 1,000 resampling.


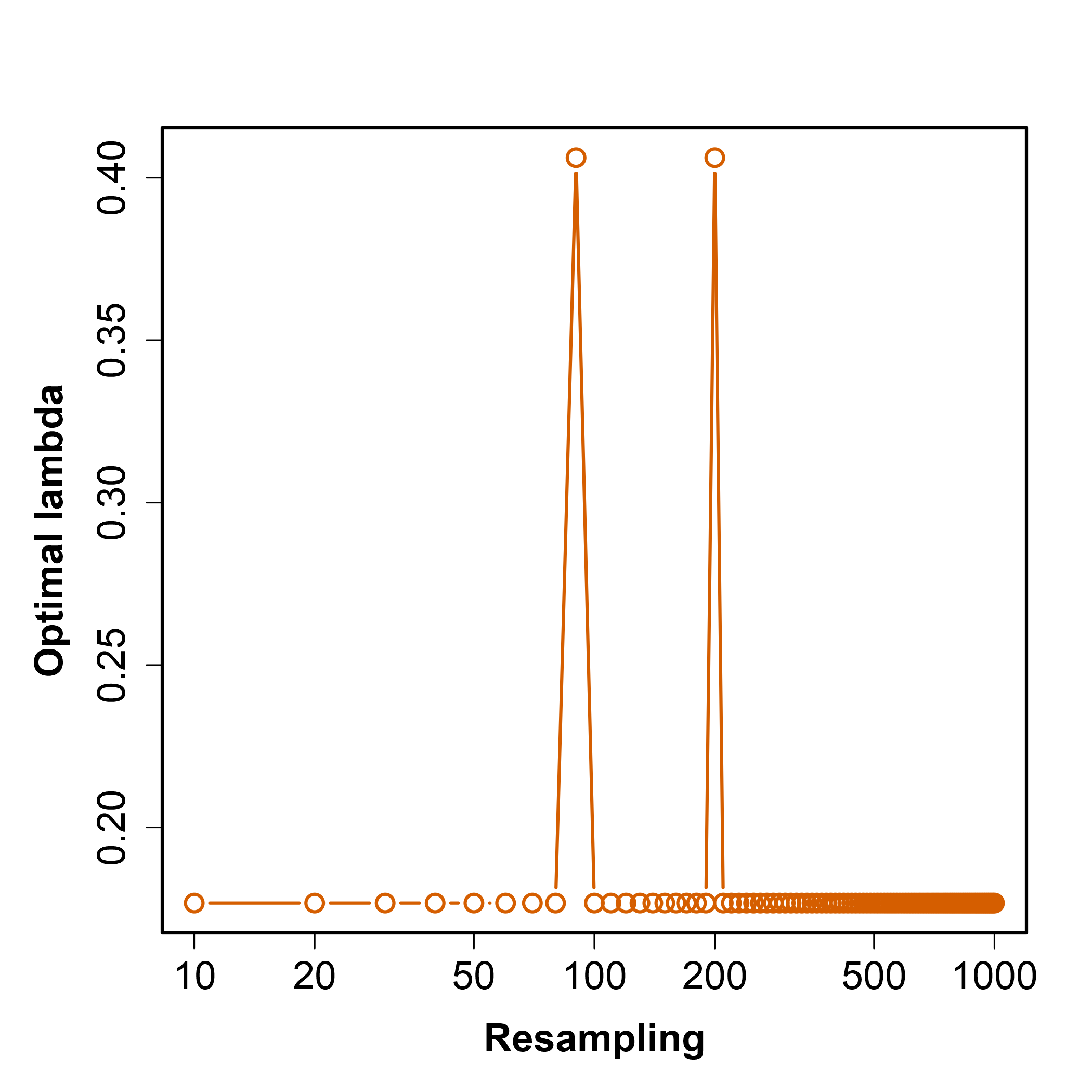


Figure S5: Convergence of model accuracy during resampling for the FNA dataset

The cumulatively estimated model accuracy during the 10,000 resampling for the elastic net model with *α=0.5* and *λ=0.177*. The accuracy after 1,000 resampling reaches 0.903.


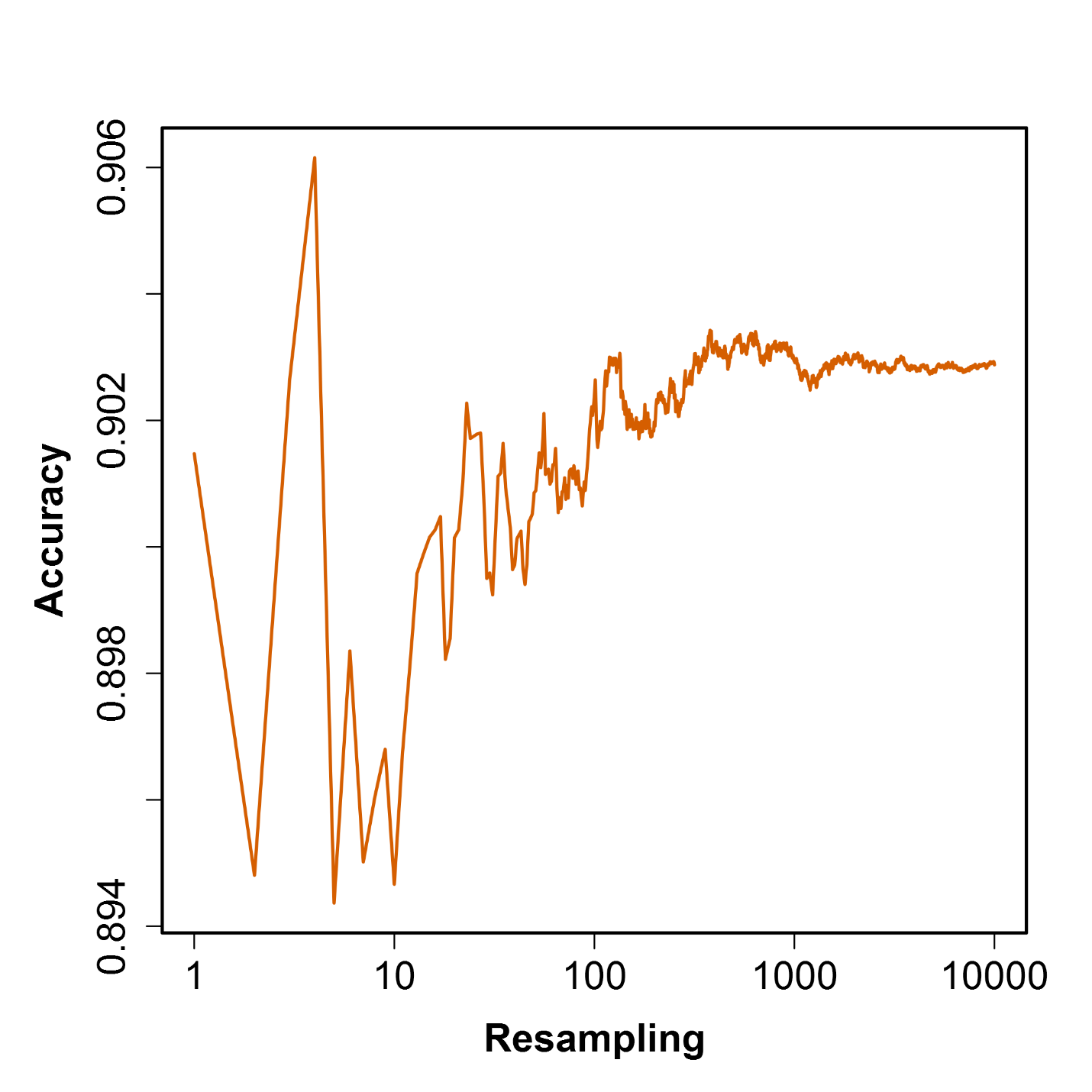


Figure S6: Model thresholds for confident subtyping in clinical implementation.

The subtyping specificity for LUAD (orange) or LUSC (green) samples varies according to subtype-specific thresholds.


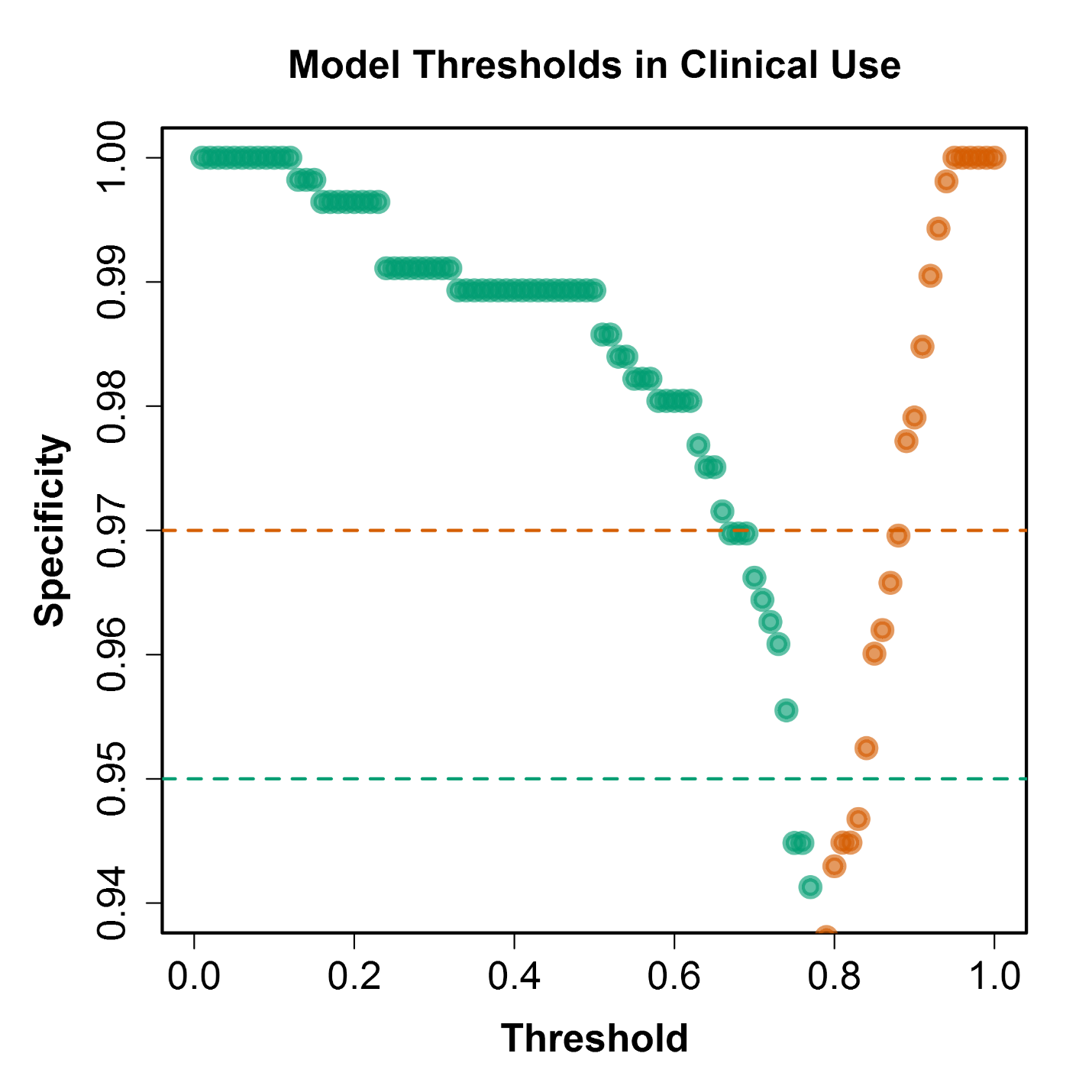


Table S1. Population characteristics of the Exploring meta-cohort.

|  | **PARIS** | **UM** | **DUKE** | **DIRECTOR’S CHALLENGE** | **Total** |
| --- | --- | --- | --- | --- | --- |
| **Patients, n (%)** | 74 (15%) | 73 (15%) | 67 (14%) | 276 (56%) | 490 |
| **GeneChip Type** | PLUS 2.0 | U133A | PLUS 2.0 | U133A | — |
| **Age, years** |  |  |  |  |  |
| **mean (SD)** | 62.6 (11.2) | 69.8 (8.7) | 65.3 (9.0) | 64.4 (10.2) | 65.1 (10.2) |
| **Histology, n (%)** |  |  |  |  |  |
| **LUAD** | 74 (100%) |  | 34 (51%) | 276 (100%) | 384 (78.4%) |
| **LUSC** |  | 73 (100%) | 33 (49%) |  | 106 (21.6%) |
| **Gender, n, %** |  |  |  |  |  |
| **Female** | 18 (24.3%) | 27 (37.0%) | 28 (41.8%) | 145 (52.5%) | 180 (46.9%) |
| **Male** | 56 (75.7%) | 46 (63.0%) | 39 (58.2%) | 131 (47.5%) | 204 (53.1%) |

UM: University of Michigan cohort; PLUS 2.0: U133 PLUS 2.0; SD: standard deviation.

Table S2: Signature genes and coefficients of the molecular subtyping model

Table S3: Performance on FNA cohort

The performance was listed below.

|  |  | **Reference (by Pathologists)** | | |  |  |
| --- | --- | --- | --- | --- | --- | --- |
|  | Total: 83 | LUAD: 47 | LUSC: 25 | NOS: 11 | **Accuracy: 93.1%** | Prevalence: 65.3% |
| **Predicted** | LUAD: 60 | 47 | 5 | 8 | Precision: 90.4% | FDR: 9.6% |
|  | LUSC: 23 | 0 | 20 | 3 |  |  |
|  | Prevalence:  100% | **Recall (Sensitivity):**  **100%** | Fall-out (FPR): 20% |  | LR+: 5 | Diagnostic Odds Ratio (DOR): inf |
|  |  | Missing Rate: 0% | **Specificity: 80%** |  | LR-: 0 |  |

Table S4: Performance on WFBH tissue bank cohort

The performance was listed below.

|  |  | **Reference (by Pathologists)** | | |  |
| --- | --- | --- | --- | --- | --- |
|  | Total: 42 | LUAD: 20 | LUSC: 22 | **Accuracy: 88.10%** | Prevalence: 47.62% |
| **Predicted** | LUAD: 25 | 20 | 5 | Precision: 80% | FDR: 20% |
|  | LUSC: 17 | 0 | 17 |  |  |
|  |  | **Recall (Sensitivity):**  **100%** | Fall-out: 22.73% | LR+: 4.4 | Diagnostic Odds Ratio (DOR): inf |
|  |  | Miss Rate: 0% | **Specificity: 77.27%** | LR-: 0 |  |

Table S5: Performance on TCGA cohort

The performance was listed below.

|  |  | **Ref (by TCGA Pathologists)** | | |  |
| --- | --- | --- | --- | --- | --- |
|  | Total: 1,016 | LUAD: 515 | LUSC: 501 | **Accuracy: 94.5%** | Prevalence: 50.7% |
| **Predicted** | LUAD: 559 | 509 | 50 | Precision: 91.1% | FDR: 8.9% |
|  | LUSC: 457 | 6 | 451 |  |  |
|  |  | **Recall (Sensitivity):**  **98.8%** | Fall-out: 10.0% | LR+: 9.9 | Diagnostic Odds Ratio (DOR): 765.2 |
|  |  | Miss Rate: 1.2% | **Specificity: 90.0%** | LR-: 0.01 |  |

Table S6: Probes on the 67-gene panel

Sample Code

A sample code for molecular-based lung cancer subtyping was provided below.

> sampleGctFile <- system.file(file.path('testdata', 'sample.gct')

, package='FNASubtype')

> gctData <- loadGctData(sampleGctFile)

> inputMatrix <- buildInputMatrix(gctData)

> predictions <- generatePredictions(inputMatrix)

> predictions

probability type

SuZKB 0.003750783 S

LpO3F 0.104243299 S

oNPzf 0.002300854 S

huWrt 0.058568001 S

Q8f1b 0.142180705 S

vITSC 0.004076386 S

CYZKk 0.138152172 S

Hen6P 0.057430369 S

lA4Aq 0.132798829 S

F219O 0.043053089 S

...

References

1. Broet P, Camilleri-Broet S, Zhang S, Alifano M, Bangarusamy D, Battistella M, Wu Y, Tuefferd M, Regnard JF, Lim E, et al: **Prediction of clinical outcome in multiple lung cancer cohorts by integrative genomics: implications for chemotherapy selection.** *Cancer Res* 2009, **69:**1055-1062.

2. Raponi M, Zhang Y, Yu J, Chen G, Lee G, Taylor JM, Macdonald J, Thomas D, Moskaluk C, Wang Y, Beer DG: **Gene expression signatures for predicting prognosis of squamous cell and adenocarcinomas of the lung.** *Cancer Res* 2006, **66:**7466-7472.

3. Bild AH, Yao G, Chang JT, Wang Q, Potti A, Chasse D, Joshi MB, Harpole D, Lancaster JM, Berchuck A, et al: **Oncogenic pathway signatures in human cancers as a guide to targeted therapies.** *Nature* 2006, **439:**353-357.

4. Director's Challenge Consortium for the Molecular Classification of Lung A, Shedden K, Taylor JM, Enkemann SA, Tsao MS, Yeatman TJ, Gerald WL, Eschrich S, Jurisica I, Giordano TJ, et al: **Gene expression-based survival prediction in lung adenocarcinoma: a multi-site, blinded validation study.** *Nat Med* 2008, **14:**822-827.

5. Chen H, Chen W, Liu C, Zhang L, Su J, Zhou X: **Relational Network for Knowledge Discovery through Heterogeneous Biomedical and Clinical Features.** *Sci Rep* 2016, **6:**29915.

6. Dotson T, Bellinger C, Su J, Hansen K, Parks GE, Cappellari JO, Craddock L, Clark H, Howard C, Petty WJ, et al: **Feasibility of lung cancer RNA acquisition from a single transbronchial or transthoracic needle pass (FASTT trial).** *Lung Cancer* 2019, **127:**6-11.

7. Guo Y, Hastie T, Tibshirani R: **Regularized linear discriminant analysis and its application in microarrays.** *Biostatistics* 2007, **8:**86-100.

8. Brown MP, Grundy WN, Lin D, Cristianini N, Sugnet CW, Furey TS, Ares M, Jr., Haussler D: **Knowledge-based analysis of microarray gene expression data by using support vector machines.** *Proc Natl Acad Sci U S A* 2000, **97:**262-267.

9. Ramaswamy S, Tamayo P, Rifkin R, Mukherjee S, Yeang CH, Angelo M, Ladd C, Reich M, Latulippe E, Mesirov JP, et al: **Multiclass cancer diagnosis using tumor gene expression signatures.** *Proc Natl Acad Sci U S A* 2001, **98:**15149-15154.

10. Dabney AR: **Classification of microarrays to nearest centroids.** *Bioinformatics* 2005, **21:**4148-4154.

11. Tibshirani R, Hastie T, Narasimhan B, Chu G: **Diagnosis of multiple cancer types by shrunken centroids of gene expression.** *Proc Natl Acad Sci U S A* 2002, **99:**6567-6572.

12. Wright GW, Simon RM: **A random variance model for detection of differential gene expression in small microarray experiments.** *Bioinformatics* 2003, **19:**2448-2455.

13. Zhao Y, Simon R: **BRB-ArrayTools Data Archive for human cancer gene expression: a unique and efficient data sharing resource.** *Cancer Inform* 2008, **6:**9-15.

14. Simon R, Lam A, Li MC, Ngan M, Menenzes S, Zhao Y: **Analysis of gene expression data using BRB-ArrayTools.** *Cancer Inform* 2007, **3:**11-17.
